# Interruption of anti-thymocyte globuline treatment in solid organ transplantation is effectively monitored through a low total lymphocyte count

**DOI:** 10.3389/fimmu.2024.1419726

**Published:** 2024-06-12

**Authors:** Rikke Olund Rasander, Søren Schwartz Sørensen, Paul Suno Krohn, Helle Bruunsgaard

**Affiliations:** ^1^ Section of Transplantation Immunology, The Tissue Typing Laboratory, Department of Clinical Immunology, Copenhagen University Hospital – Rigshospitalet, Copenhagen, Denmark; ^2^ Department of Nephrology, Copenhagen University Hospital – Rigshospitalet, Copenhagen, Denmark; ^3^ Department of Clinical Medicine, University of Copenhagen, Copenhagen, Denmark; ^4^ Department of Surgery and Transplantation, Copenhagen University Hospital – Rigshospitalet, Copenhagen, Denmark

**Keywords:** anti-thymocyte globuline, ATG, transplantation, T-lymphocytes, lymphocytes, monitoring, kidney, pancreas

## Abstract

**Introduction:**

Anti-Thymocyte Globulin (ATG) is a cornerstone in immune suppression for solid organ transplantation. The treatment is a delicate balance between complications arising from over-immunosuppression such as infections and cancer versus rejection stemming from under-immunosuppression. CD3^+^ T-lymphocyte measurements are frequently employed for treatment monitoring. However, this analysis is costly and not always accessible. The aim of this study was to investigate whether the total count of lymphocytes could replace CD3^+^ T-lymphocyte measurements based on data from our transplantation center combined with a review of the literature. The hypothesis was that the total lymphocyte count could serve as a diagnostic surrogate marker for CD3^+^ T-lymphocytes.

**Methods:**

A retrospective cohort study was conducted, including patients who underwent kidney and/or a pancreas transplantation and received ATG as induction therapy or for rejection treatment. The inclusion criterium was that the total lymphocyte count and CD3^+^ T-lymphocyte measurements were measured simultaneously on the same day. Additionally, PubMed and Embase were searched up to 18/10/2023 for published studies on solid organ transplantation, ATG, T-lymphocytes, lymphocyte count, and monitoring. In the retrospective cohort study, a total of 91 patients transplanted between 2016 and 2023, with 487 samples, were included.

**Results:**

Total lymphocyte counts below 0.3 x 10^9^/L had a high sensitivity (86%) as a surrogate marker of CD3^+^ T-lymphocytes below 0.05 x 10^9^/L, but the specificity was low (52%) for total lymphocyte counts above 0.3 x 10^9^/L as a surrogate marker for CD3^+^ T-lymphocytes above 0.05 x 10^9^/L. A review of the literature identified seven studies comparing total lymphocyte counts and CD3^+^ T-lymphocytes in ATG monitoring. These studies supported the use of a low total lymphocyte count as a surrogate marker for CD3^+^ T-lymphocytes and an indicator to omit ATG treatment. However, there was no consensus regarding high total lymphocyte counts as an indicator for continued treatment.

**Discussion:**

Results supports that the total lymphocyte count can be used to omit ATG treatment when below 0.3 x 10^9^/L whereas the CD3^+^ T-lymphocyte analysis should be reserved for higher total lymphocyte counts to avoid ATG overtreatment.

## 1 Introduction

Anti-Thymocyte Globulin (ATG) is a purified immunoglobulin (Ig) G antibody produced in rabbits or horses after immunization with human thymus cells (1). ATG functions as a T-lymphocyte depletor with affinity for various lymphocyte receptors, reducing the number of circulating lymphocytes through complement-dependent lysis, T-cell apoptosis, and opsonization (2). One of the primary uses of ATG is to suppress the immune system during transplantation to prevent rejection or to treat severe acute rejection. The treatment is a delicate balance between complications arising from over-immunosuppression, such as infections and cancer, versus rejection stemming from under-immunosuppression. Additionally, ATG treatment can cause life-threatening cytokine-release syndrome (3). Therefore, it has been recommended that ATG dosing is tailored to the individual patient by measurements of CD3^+^ T-lymphocytes (4–6).

In our transplantation center, ATG is administered as an induction therapy for high-risk immunological patients undergoing kidney transplantation, for all simultaneous kidney-pancreas transplantations and pancreas-after-kidney transplantation, and in some cases of rejection treatment of kidney transplant recipients. Currently, ATG dosage adjustment relies on the measurement of CD3^+^ T-lymphocytesa using flowcytometry. When CD3^+^ T-lymphocyte counts fall below 0.05 x 10^9^/L, ATG treatment is paused. This approach is supported by strong evidence from consistent reports, indicating that CD3^+^ T-lymphocyte counts below 0.02–0.05 x 10^9^/L are sufficient to protect against rejection (7–10), and individual monitoring can help prevent infections and over treatment (4–6, 11, 12). However, in many clinical settings, CD3^+^ T-lymphocyte measurements by flowcytometry are typically performed only during weekday daytime hours in specialized laboratories. Outside of these hours, the simpler, more feasible, and cost-effective leukocyte and differential count is used, with a threshold of < 0.3 x 10^9^/L total lymphocytes to determine when to pause treatment.

The aim of this study was to investigate whether the easily accessible and cost-effective measurement of total lymphocyte count could replace CD3^+^ T-lymphocyte measurements in the therapeutic monitoring of ATG based on retrospective data spanning a seven-year period at our transplantation center. The findings were combined with a review of the current literature to evaluate and optimize procedures and recommendations for dosing ATG treatment in solid organ transplantation. The hypothesis was that total lymphocyte counts below 0.3 x 10^9^/L would have a high predictive value for CD3^+^ T-lymphocyte counts below 0.05 x 10^9^/L, which would trigger the pausing of ATG treatment. Additionally, it was hypothesized that total lymphocyte counts above 0.3 x 10^9^/L would be a good predictor of CD3^+^ T-lymphocyte levels above 0.05 x 10^9^/L, leading to the continuation of ATG treatment. A systematic review of the literature was conducted in parallel.

## 2 Materials and methods

### 2.1 Retrospective cohort study

The retrospective study cohort consisted of patients who underwent kidney transplantation, simultaneous kidney-pancreas transplantation, and pancreas-after-kidney transplantation at Copenhagen University Hospital – Rigshospitalet, Copenhagen. The inclusion criteria were patients who received ATG as induction (prophylactic) therapy, or patients where ATG was used to treat rejection episodes during the period from March 11, 2015, to September 1, 2023. Patients were excluded if simultaneous measurements of CD3^+^ T-lymphocytes and total lymphocyte counts were not available during the ATG treatment period.

#### 2.1.1 Total lymphocyte counts and CD3^+^ T-lymphocytes

The total lymphocyte count was measured as part of an automated total leukocyte count, which includes a group count covering lymphocytes, monocytes, neutrophils, eosinophils, and basophils, at the Department of Biochemistry, Copenhagen University Hospital - Rigshospitalet.

The concentration of CD3^+^ T-lymphocytes was measured using flowcytometry as part of a volumetric identification analysis. This analysis includes testing for CD45, CD3, CD4, and CD8 subpopulations in one tube, and CD45, CD19, CD16, and CD56 in another tube, using an AQUIOS instrument (Beckman Coulter) at the Department of Clinical Immunology, Copenhagen University Hospital – Rigshospitalet. Both the total lymphocyte count and the flow cytometry measurements of lymphocyte subpopulations are accredited analyses according to ISO 15189 standards.

#### 2.1.2 Data sources

Clinical data and laboratory data were extracted from the clinical patient record and associated laboratory systems.

#### 2.1.3 Statistical analyses

Statistical analyses were conducted using IBM SPSS Statistics 22 and RStudio. The distribution of total lymphocyte counts and CD3^+^ T-lymphocytes were left-skewed. Therefore, Spearman correlations were employed to assess associations between these variables. CD3^+^ T-lymphocytes below 0.05 x 10^9^/L served as the true value/golden standard for discontinuation of ATG treatment, while a total lymphocyte count <0.3 x 10^9^/L was considered a diagnostic surrogate marker, encompassing true positives, false positives, false negatives, and true negatives. Sensitivity, specificity, positive predictive values, and negative predictive values were calculated based on this categorization within a 2x2 confusion matrix. Additionally, a Receiver Operating Characteristic (ROC) curve was generated to determine the statistical optimal threshold of the total lymphocyte count for distinguishing between CD3^+^ T-lymphocyte counts below or above 0.05 x 10^9^/L. The Youden index, J, was utilized to identify the most optimal threshold, calculated as J = (true positives)/(true positives + false negatives) + (true negatives)/(true negatives + false positives) - 1.

#### 2.1.4 Ethical considerations

The study was approved by the secretariat for the local scientific ethical committee, Center for Health, Capital Region, Denmark (R-23050332 and J-23056078), which serves as the local Danish patient safety authority, and the local Danish data protection authority (p-2023–14569).

### 2.2 Systematic review

A review was conducted in adherence with the recommendations in the Preferred Reporting Items for Systematic Reviews and Meta-Analyses (PRISMA) statement (13, 14) summarized in **Supplementary Table 1**.

The review included studies of patients treated with ATG (intervention) in relation to solid organ transplantation or treatment of a rejection episode after solid organ transplantation, and where CD3^+^ T-lymphocytes were compared with total lymphocyte counts in monitoring of the ATG treatment. Exclusion criteria were animal studies, transplantation studies without ATG treatment, and conference abstracts. National Library of Medicine Database and Embase were searched up to 18/10/2023. Subject headings and text words were used with related terms for “Transplantation”, “CD3^+^ T-lymphocytes”, “Total lymphocyte count”, “monitoring”, and “ATG” (**Supplementary Tables 2**, **3**). COVIDENCE (https://www.covidence.org/) was used to screen studies and to decide on inclusion/exclusion.

## 3 Results

### 3.1 Retrospective cohort study

**Figure 1:**
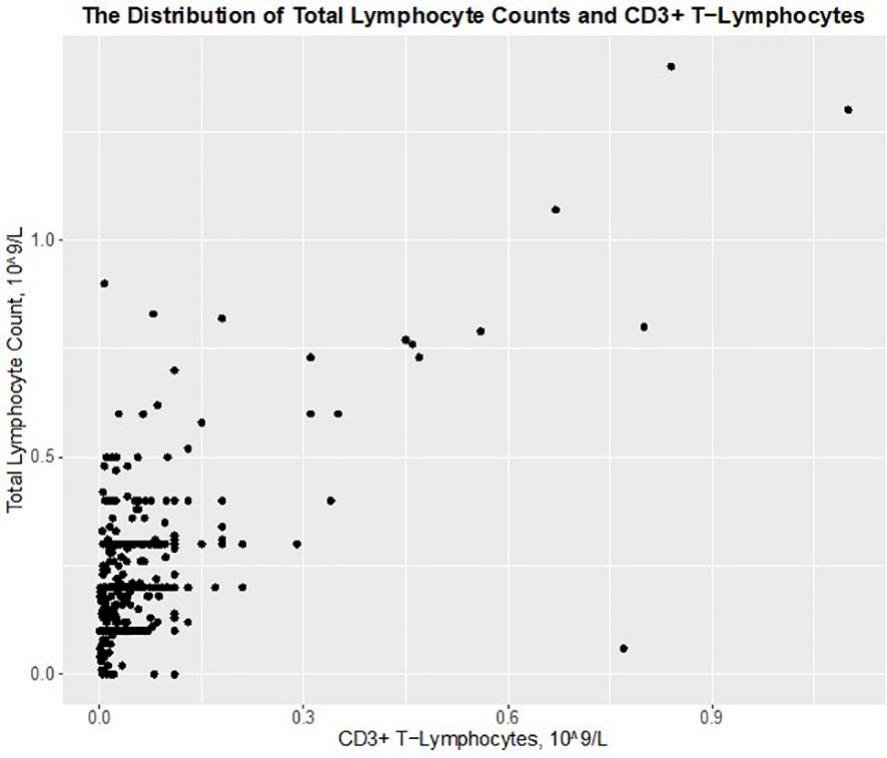
The distribution of total lymphocyte counts and CD3^+^ T–lymphocytes in 91 ATG treated transplant recipient with 487 simultaneous measurements.

In the period from March 11, 2015, to September 1, 2023, we identified 58 kidney transplantations, 42 simultaneous kidney-pancreas transplantations, and three pancreas-after-kidney transplantations receiving ATG induction/prophylactic therapy, totaling 103 patients. Additionally, 18 kidney transplant patients were treated with ATG due to rejection. Thirty patients were excluded because simultaneous measurements of total lymphocytes counts and CD3^+^ T-lymphocyte counts were not available. This left us with 91 patients for study, with a median of 4 simultaneous measurements (range 1–12), totaling 487 simultaneous measurements of total lymphocyte counts and CD3^+^ T-lymphocyte counts. The distribution of these simultaneous measurements is shown in **Figure 1**, with an overweight of measurements showing low levels of CD3^+^ T-lymphocytes and total lymphocyte counts, as expected. A moderate Spearman correlation was found between CD3^+^ T-lymphocytes and total lymphocyte count: R_S_ = 0.521, N = 487, p < 0.001.

The data were divided into groups based on total lymphocyte counts < 0.3 x 10^9^/L and ≥ 0.3 x 10^9^/L versus CD3^+^ T-lymphocytes < 0.05 x 10^9^/L and ≥ 0.05 x 10^9^/L (**Table 1**). With these thresholds, total lymphocyte counts less than 0.3 x 10^9^/L had a sensitivity of 85% and a positive predictive value of 84% as a surrogate marker of CD3^+^ T-lymphocytes < 0.05 x 10^9^/L. However, a total lymphocyte count ≥ 0.3 x 10^9^/L had a poor specificity of 52% and a low negative predictive value of 57% for CD3^+^ T-lymphocytes ≥ 0.05 x 10^9^/L.

**Table 1:** 2x2 Matrix of the total lymphocyte count against CD3^+^ T–lymphocytes.

	CD3^+^ T–lymphocytes < 0.05 mia/L	CD3^+^ T–lymphocytes ≥ 0.05 mia/L
**Total lymphocyte count** **< 0.3 mia/L**	N = 309 (86 % = *sensitivity*) True positive	N = 61 (14 %) False positive
**Total lymphocyte count** **≥ 0.3 mia/L**	N = 50 (48 %) False negative	N = 67 (52 %= *specificity*) True negative

**Figure 2:**
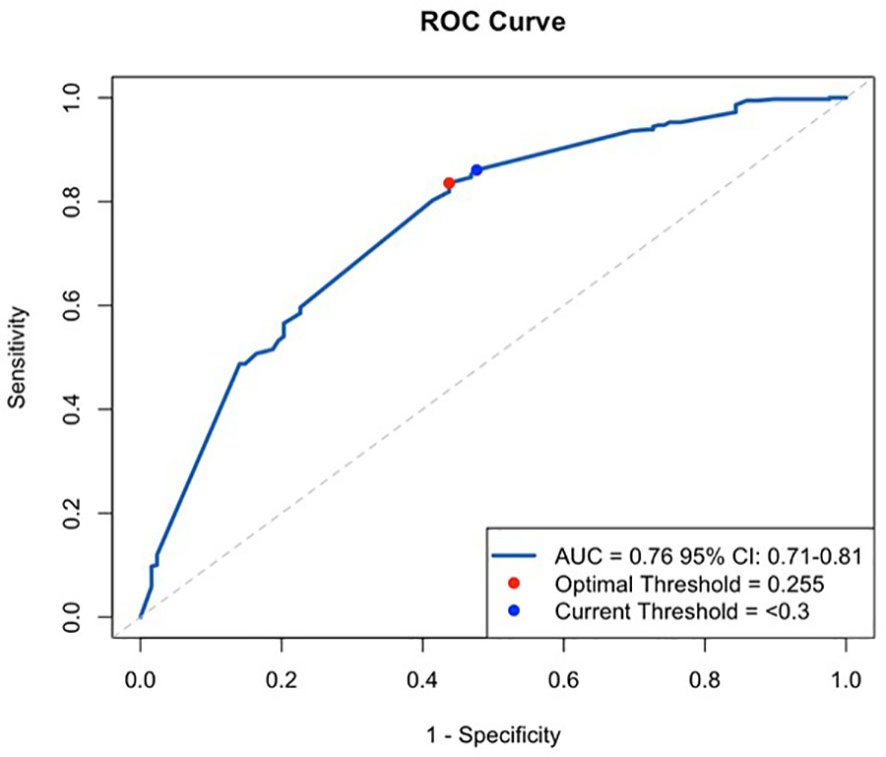
Receiver Operating Characteristic, ROC curve, with current threshold at 0.3 10^9^/L total lymphocytes and the statistically optimal threshold at 0.255 10^9^/L.

A ROC curve was constructed to test the statistical optimal threshold for the total lymphocyte count to be a diagnostic test of CD3^+^ T-lymphocytes < 0.05 x 10^9^/L. (**Figure 2**). The statistically optimal threshold was found to be 0.255 x 10^9^/L, which is equivalent to 0.3 x 10^9^/L in the clinical setting.

It was explored whether the poor specificity and low negative predictive value of a total lymphocyte count were caused by contributions from NK cells and B-lymphocytes (**Table 2**). The sum of these two cell populations contributed to a median of 75.4% of the total lymphocytes, with a minimum of 8.4% with a maximum of 100%.

**Table 2:** Comparisons of CD3+ T–lymphocytes and total lymphocyte counts in monitoring of ATG treatment in kidney transplantation.

Study	Number of patients	Threshold for interrupting ATG treatment	Diagnostic value of the total lymphocyte count
Retrospective cohort study	20 patients	CD3^+^ T–lymphocytes 0.02 x 10^9^/L Total lymphocytes 0.2 x 10^9^/L	R=0.9, p<0.0001 Total lymphocytes < 0.2 x 10^9^/L is 92.4% predictive of CD3^+^ T–lymphocytes < 0.02 x 10^9^/L
Prospective cohort study	8 patients: Induction = 1 Rejection = 7	CD3^+^ T–lymphocytes 0.02–0.05 x 10^9^/L Total lymphocytes 0.3 x 10^9^/L	High predictive value of total lymphocytes < 0.3 x 10^9^/L and CD3^+^ T–lymphocytes < 0.05 x 10^9^/L Poor predictive value (60%) of total lymphocytes > 0.3 x 10^9^/L and CD3^+^ T–lymphocytes <0.05 x 10^9^/L
Retrospective cohort study	76 paired samples	CD3^+^ T–lymphocytes 0.05 x 10^9^/L Total lymphocytes 0.1 x 10^9^/L or 0.2 x 10^9^/L	68% discrepancy in clinical decisions
Prospective cohort study	24 patients: Induction = 14 Rejection = 10 298 paired samples	CD3^+^ T–lymphocytes 0.01 x 10^9^/L Total lymphocytes 0.1 x 10^9^/L	Rs=0.4, P<0.001 66% discrepancy in clinical decisions 23% overtreatment
Retrospektive cohort study	242 patients	CD3^+^ T–lymphocytes 0.02 x 10^9^/L	Correlation between CD3^+^ T–lymphocytes and total lymphocytes R=0.88, p<0.001 Total lymphocyte count >0.07 x 10^9^/L had 68% sensitivity of CD3^+^ T–lymphocytes > 0,02 x 10^9^/L and 69% specificity (ROC curve analysis).
Retrospektivt kohortestudie	226 patients Induction = 201 Rejection = 25 664 paired samples	CD3^+^ T–lymphocytes 0.02 x 10^9^/L	Correlation between CD3^+^ T–lymphocytes and total lymphocytes R=0.42, p<0.001 Total lymphocyte count 0.256 x 10^9^/L had 67% sensitivity of CD3^+^ T–lymphocytes < 0.02 x 10^9^/L and 67% specificity (ROC curve analysis) .

### 3.2 Systematic literature review

From an initial search and screening, 64 relevant references were identified. Among these, only six studies compared the total lymphocyte count and CD3^+^ T-lymphocyte levels in monitoring ATG treatment, comprising two prospective and four retrospective cohort studies (**Table 2**).

Throughout all studies, CD3^+^ T-lymphocyte concentrations were universally regarded as the golden standard for guiding ATG treatment, although the specific threshold for pausing varied within the range of 0.01**–**0.05 x 10^9^/L (15–20). Most studies reported a correlation ranging from weak to strong between CD3^+^ T-lymphocyte levels and total lymphocyte counts, particularly notable for lower values of total lymphocyte counts, while concerns arose regarding the potential influence of B-lymphocytes at higher total lymphocyte counts (15–20).

As a diagnostic tool, a total lymphocyte count below 0.2–0.3 x 10^9^/L was consistently associated with a CD3^+^ T-lymphocyte levels < 0.02–0.05 x 10^9^/L, demonstrating good sensitivity and positive predictive value (15, 16). There was consensus among the two studies that treatment solely based on the total lymphocyte count below 0.2–0.3 x 10^9^/L posed a risk of undertreatment in 3–8% of patients (15, 16).

Conversely, regarding a total lymphocyte count exceeding 0.1**–**0.3 x 10^9^/L, consensus on diagnostic value was lacking when evaluating specificity and prediction of a CD3^+^ T-lymphocyte level surpassing 0.02**–**0.1 x 10^9^/L. One study identified a poor predictive value, suggesting a risk of overtreatment (16) while two studies reported acceptable specificity and predictive value (19, 20). One study solely focused on the sensitivity and predictive value of a low total lymphocyte count (15), and two studies addressed the overall discordance between the two methods but did not specifically evaluate the diagnostic value of a low total lymphocyte count (17, 18), reporting either overtreatment in 23% of cases (18) or leaving the evaluation unaddressed (17). Only two studies delineated the optimal cutoff value for the total lymphocyte count to balance sensitivity and specificity as a diagnostic test for CD3^+^ T-lymphocyte levels (19, 20). However, despite both studies using CD3^+^ T-lymphocytes at 0.02 x 10^9^/L as a reference, the reported threshold for total lymphocyte counts varied from 0.07 x 10^9^/L (19) to 0.256 x 10^9^/L (20). Total lymphocyte counts greater than 0.07 x 10^9^/L had a 68% sensitivity of CD3^+^ T-lymphocytes greater than 0,02 x 10^9^/L (19). This indicates the optimal performance of the diagnostic test for deciding when to continue ATG administration based on total lymphocyte counts and not to interrupt the treatment, as demonstrated in our study and (20).

## 4 Discussion

This study offers the largest data set in the literature to evaluate the diagnostic value of both a low and a high total lymphocyte count in ATG administration, compared to the gold standard, which is the number of CD3^+^ T-lymphocytes. It encompasses 91 patients, and 487 simultaneous measurements of total lymphocyte counts and CD3^+^ T-lymphocytes. It is firmly established in the literature by controlled randomized (12) and controlled non-randomized studies (5, 6, 10) that maintaining CD3^+^ T-lymphocyte levels below 0.05 x 10^9^/L serves as a robust indicator for safely reducing ATG treatment, balancing the risks of rejection and infections. The primary finding from our retrospective cohort study at the Copenhagen Transplantation Centre underscores the effectiveness of utilizing an easily accessible total lymphocyte count below 0.3 x 10^9^/L as a reliable diagnostic surrogate marker for low CD3^+^ T-lymphocytes, prompting the cessation of ATG treatment. Conversely, relying on a higher total lymphocyte count than 0.3 x 10^9^/L to guide ongoing ATG treatment poses a significant risk of overtreatment, as nearly half of the measurements would have CD3^+^ T-lymphocyte levels below the threshold of 0.05 x 10^9^/L. Our study also provides supporting evidence that the poor specificity of high total lymphocyte counts as a diagnostic surrogate marker of the CD3^+^ T-lymphocyte concentration may be attributed to significant contributions from NK cells and B-lymphocytes, a hypothesis previously suggested but not conclusively demonstrated (15, 17). Our findings advocate for a monitoring strategy utilizing a total lymphocyte count below 0.3 x 10^9^/L to guide reductions in ATG treatment. Supplementary measurements of CD3^+^ T-lymphocyte concentration could be reserved for instances where total lymphocyte counts are higher than 0.3 x 10^9^/L, aiming to avoid unnecessary treatment. This conclusion aligns with observations and recommendations put forward by Gorrie et al. (16). This study (16) is the only previously published study that evaluates both the diagnostic value of a low and high total lymphocyte counts and make a clinical distinction between these two clinical scenarios. Notably, while the study by Gorrie et al. (16) was based on a limited cohort of 8 patients, our study contributes with more substantial data to bolster this proposed strategy. Buchler et al. (15) additionally noted that a low total lymphocyte count exhibits high sensitivity as a surrogate marker for a low CD3^+^ T-lymphocyte level, warranting attention to ATG treatment adjustments in their study involving 20 patients. However, the Buchler et al. (15) study did not delve into the specificity of higher total lymphocyte counts due to the relevant concerns regarding the potential influence of B-lymphocytes documented by our data.

Our review of the literature showed that studies use thresholds for the total lymphocyte count between 0.07–0.3 x 10^9^/L as a diagnostic surrogate test of the CD3^+^ T-lymphocyte level (15–20). We attempted in our retrospective cohort study to find the optimal threshold to balance between sensitivity and specificity by the construction of a ROC curve, demonstrating 0.255 x 10^9^/L to be statistically optimal in the middle of the commonly used interval in other studies. In accordance with this, Machado et al. (20) reported 0.256 x 10^9^/L to be the threshold to predict the CD3^+^ T-lymphocyte level to guide ATG treatment but the reference concentration for the CD3^+^ T-lymphocyte levels was 0.02 x 10^9^/L in their study. Differences in the applied threshold of CD3^+^ T-lymphocyte levels between Machado et al. (20) and our retrospective cohort study is likely to explain that Machado et al. (20) found a lower sensitivity and a higher specificity than our retrospective cohort study. Buchler et al. (15) found a low total lymphocyte count to be a better predictor of a low CD3^+^ T-lymphocyte concentration compared to our study but both thresholds for total lymphocyte counts and CD3^+^ T-lymphocyte levels were lower, and a ROC curve was not constructed. It is unclear why the construction of a ROC curve in the study by Furlanetto et al. (19) resulted in a threshold of total lymphocyte counts to be 0.07 x 10^9^/L, that is much lower compared to our results and Machado et al. (20) although their reference CD3^+^ T-lymphocyte level (19) was equal to Machado et al. (20). If the true absolute lymphocyte count threshold as a surrogate to CD3^+^ T-lymphocytes is closer to 0.07 than 0.3 x 10^9^/L, then there is a risk of undertreating patients who would otherwise benefit from ATG. Such a risk will always be a part of a diagnostic test and a sensitivity of 86% in our study is considered good and in accordance with reports in two other studies (15, 16). Additionally, Furlanetto et al. (19) examined the sensitivity of the total lymphocyte count in predicting a higher CD3+ T-lymphocyte level and continued ATG administration. Given this objective, it is anticipated that the threshold would be lower. It is very likely that the reported high discrepancy in clinical decisions based on the total lymphocyte count versus the CD3^+^ T-lymphocyte level (17, 18) is caused by the poor predictive value of higher total lymphocyte counts demonstrated in our study and by Gorrie et al. (16) as (17, 18) did not explore the diagnostic value of a low total lymphocyte count.

In clinical practice, a strategy involving sequential measurements of total lymphocytes, followed by analyses of CD3^+^ T-lymphocytes concentration, may delay an interruption of unnecessary administration of ATG treatment in cases with high total lymphocyte counts. We recommend that in cases with higher total lymphocyte counts, ATG treatment should be continued until the CD3+ T-lymphocyte measurements are obtained. Exceptions should be considered in cases where side effects or clinical circumstances favor rapid intervention without cost concerns or logistical laboratory problems. It is also important to note that many solid organ transplantation programs do not currently monitor ATG treatment at all, so implementing total lymphocyte count monitoring will be an added benefit.

Our study did not explore the optimal time duration of a low CD3+ T-lymphocyte level or a low total lymphocyte count in ATG induction therapy or rejection treatment and it was not evaluated in the studies, which our literature review was based on. It could be considered as a limitation that the 487 simultaneous measurements of total lymphocyte counts and CD3+ T-lymphocyte counts were not independent of each other as each of the 91 patients participated with median 4 measurements. However, this is a common practice in diagnostic tests also reflected in the literature review. The study’s retrospective design limits the ability to establish causality and control for all confounding variables. Potential confounders in the study include patient demographics such as age, sex, and ethnicity, which can affect immune responses and lymphocyte counts. Additionally, comorbidities, concurrent medications, transplant characteristics (including the transplanted organ, time since transplantation, and prior episodes of rejections), and laboratory variability are other factors that might influence lymphocyte counts and responses to ATG treatment. Furthermore, there may be clinical situations where CD3^+^ T-lymphocyte counts exceed 0.05 x 10^9^/L even when the absolute lymphocyte count is below 0.3 x 10^9^/L. While serum sickness is a potential concern, it is expected to present with additional symptoms. Some patients were excluded due to the lack of simultaneous measurements of total lymphocyte and CD3^+^ T-lymphocyte counts. Variability in the timing of lymphocyte counts and potential delays between clinical decision-making and actual measurements could introduce bias. Moreover, the study population was limited to a specific transplant center, which may not be representative of all transplant recipients. Variations in ATG dosing regimens and monitoring protocols across different centers could affect the generalizability of the findings.

In conclusion, the retrospective cohort study and systematic literature review collectively contribute to our understanding of the role of total lymphocyte counts in monitoring ATG therapy. While low total lymphocyte counts act as a good surrogate marker for low levels of CD3^+^ T-lymphocytes in ATG monitoring, higher levels is a poor predictor caused by the contribution from other lymphocyte subsets such as B-lymphocytes and NK cells. A total lymphocyte count below 0.3 x 10^9^/L can therefore be used to omit ATG treatment but not to guide a continued administration at higher levels.

## Data availability statement

The original contributions presented in the study are included in the article/**Supplementary material**, further inquiries can be directed to the corresponding author/s.

## Ethics statement

The secretariat for the local scientific ethical committee, Center for Health, Capital Region, Denmark (R-23050332 and J-23056078), which serves as the local Danish patient safety authority, and the local Danish data protection authority (p-2023-14569). The studies were conducted in accordance with the local legislation and institutional requirements. The ethics committee/institutional review board waived the requirement of written informed consent for participation from the participants or the participants' legal guardians/next of kin because Danish law permits retrospective studies of clinical laboratory data obtained as a part a standard treatment without written informed consent if it has been approved by the health authorities for each study.

## Author contributions

RR: Investigation, Writing – original draft, Writing – review & editing, Formal analysis, Visualization. SS: Conceptualization, Data curation, Writing – review & editing. PK: Conceptualization, Data curation, Writing – review & editing. HB: Conceptualization, Investigation, Methodology, Writing – original draft, Writing – review & editing, Project administration, Resources, Supervision.
