# Supplementary material for: Interruption of anti-thymocyte globuline treatment in solid organ transplantation is effectively monitored through a low total lymphocyte count
Source: Front Immunol. 2024 Jun 12;15:1419726. doi: 10.3389/fimmu.2024.1419726 (PMC11199384; doi:10.3389/fimmu.2024.1419726)
Supplement: Supplementary file 1 [file DataSheet_1.docx]

# Supplementary Material


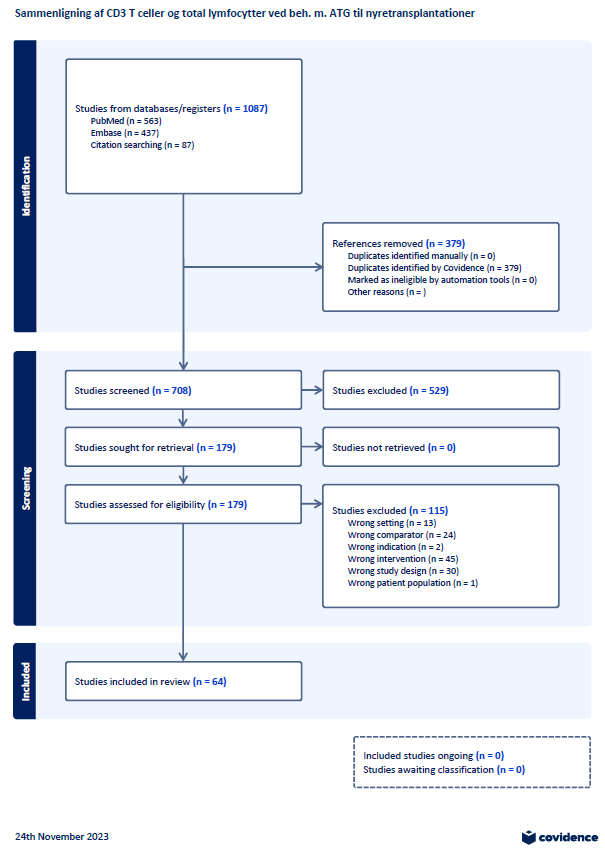
Supplementary table 1. PRISMA.

**Supplementary table 2. Construction of the search in the National Library of Medicine Database.**

|  | **Aspect 1** | **Aspect 2** | **Aspect 3** | **Aspect 4** | **Aspect 5** | **Aspect 6** |
| --- | --- | --- | --- | --- | --- | --- |
| **Subject headings** | **Transplantation** | **CD3 T lymphocytes** | **Total lymphocyte number** | **Monitoring** | **Correlation**  **(not included)** | **ATG** |
| **MeSH terms, Emtree terms, Descriptors** | Kidney transplantation  Transplants  Pancreas transplantation  Heart transplantation  Liver transplantation  Lung transplantation | T-lymphocytes  CD3 Complex  Thymocytes | Lymphocyte Count | Cytological Techniques  Flow Cytometry  Monitoring, immunologic | Correlation of data | Antilymphocyte Serum  Immunotherapy |
| **Free-text terms** | Renal transplantations  Pancreas transplantation  Heart transplantation  Liver transplantation  Lung transplantation  Grafting, heart  Grafting, liver  Grafting, lung  Grafting, pancreas  Pancreas grafting  Grafting, kidney  Kidney grafting  Kidney transplantation  Transplant  Grafts  Organ Transplant  Graft, organ | T lymphocytes  T-lymphocyte  T-cell  Thymus dependent lymphocyte  Thymocytes  CD3+  CD3  CD3 Complex | Lymphocyte Count  Lymphocyte number  Total lymphocyte count  Total lymphocyte  Lymphocyte count, total  Absolute lymphocytes | Monitoring  Monitored  Flow cytomerty  Cell monitoring  Cytologic technique  Cytologic technics  Immunologic monitoring  Immune monitoring  Monitoring, immunological | Correlation | ATG  Antilymphocyte Serum  Anti-thymocyte globulin  ATGAM  Anti-thymoglobulin  Immunotherapy  Immunosupressive therapy  Thymoglobulin |

**Supplementary table 3. Construction of the search in Embase.**

|  | **Aspect 1** | **Aspect 2** | **Aspect 3** | **Aspect 4** | **Aspect 5** | **Aspect 6** |
| --- | --- | --- | --- | --- | --- | --- |
| **Subject headings** | **Transplantation** | **CD3 T lymphocytes** | **Total lymphocyte tal** | **Monitoring** | **Correlation**  **(not included)** | **ATG** |
| **MeSH terms, Emtree terms, Descriptors** | Kidney transplantation  Transplants  Pancreas transplantation  Heart transplantation  Liver transplantation  Lung transplantation | T-lymphocytes  Thymocytes | Lymphocyte Count | Flow Cytometry  Immunologic monitoring | Correlation of data | Thymocyte antibody  Immunotherapy |
| **Free-text terms** | Renal transplantations  Pancreas transplantation  Heart transplantation  Liver transplantation  Lung transplantation  Kidney transplantation  Grafts  Grafting  Organ Transplantation | T lymphocytes  T-lymphocyte  T-cell  Thymus dependent lymphocyte  Thymocytes  CD3+  CD3  CD3 Complex | Lymphocyte Count  Lymphocyte number  Total lymphocyte count  Total lymphocyte  Lymphocyte count, total  Absolute lymphocytes | Monitoring  Monitored  Cell monitoring  Cytologic technique  Cytologic technics  Immunologic monitoring  Immune monitoring | Correlation | ATG  Antilymphocyte Serum  Anti-thymocyte globulin  ATGAM  Anti-thymoglobulin  Immunotherapy  Immunosupressive therapy  Thymoglobulin |
